# Molecular Genetic and Biochemical Characterization of Hyperphenylalaninemia Based on Expanded Neonatal Screening Data from 2023 to 2024 in the Russian Federation

**DOI:** 10.3390/ijms27125597

**Published:** 2026-06-21

**Authors:** Ekaterina E. Lotnik, Alena L. Chukhrova, Nina V. Ryadninskaya, Varvara A. Kadnikova, Ekaterina Y. Zakharova, Galina V. Baydakova, Andrey R. Osadchii, Inga V. Anisimova, Sergei V. Voronin, Sergey I. Kutsev, Kirill V. Savostyanov, Fanil S. Bilalov, Alexander L. Koroteev, Dmitry Y. Trofimov, Tatyana A. Bairova, Gulnara N. Seitova, Sergei V. Mordanov, Svetlana A. Matulevich, Tatyana A. Golikhina, Elena B. Nikolaeva, Aleksander V. Polyakov, Olga A. Shchagina

**Affiliations:** 1 Research Centre for Medical Genetics, Moskvorechie Str., 1, 115522 Moscow, Russia; achukhrova@yandex.ru (A.L.C.); outremal@yandex.ru (N.V.R.); vkadnikova@gmail.com (V.A.K.); labnbo@med-gen.ru (E.Y.Z.); baydakova@med-gen.ru (G.V.B.); oca_98@mail.ru (A.R.O.); anisimova-inga@med-gen.ru (I.V.A.); voronin.sv@med-gen.ru (S.V.V.); kutsev@mail.ru (S.I.K.); apol@dnalab.ru (A.V.P.); schagina@med-gen.ru (O.A.S.); 2 National Medical Research Centre for Children’s Health, Lomonosovskiy Prospekt, 2 b.1, 119991 Moscow, Russia; 7443333@gmail.com; 3 Republican Medical Genetic Centre, Gafuri St., 74, 450076 Ufa, Russia; bilalov@bk.ru; 4 Diagnostic Centre (Medical Genetic), Tobolskaya St., 5A, 194044 Saint-Petersburg, Russia; alexkoroteev@mail.ru; 5 National Medical Research Center for Obstetrics, Gynecology and Perinatology Named After Academician V.I. Kulakov, Akademika Oparina St., 4, 117997 Moscow, Russia; molgen@bk.ru; 6 Scientific Centre for Family Health and Human Reproduction Problems, Timiryazeva St., 16, 664003 Irkutsk, Russia; tbairova38@mail.ru; 7 Tomsk National Research Medical Centre of the Russian Academy of Sciences, Naberezhnaya Reki Ushaiki St., 10, 634050 Tomsk, Russia; gulnara.seitova@medgenetics.ru; 8 Laboratory Department of the Medical Genetic Center, Rostov State Medical University, Nakhichevanskiy per., 29, 344022 Rostov-na-Donu, Russia; labmed@mail.ru; 9 Scientific Research Institute-Ochapovsky Regional Clinical Hospital No. 1 of the Ministry of Health of the Krasnodar Region, No. 167, 1 Maya St., 350086 Krasnodar, Russia; s.matulevich@yandex.ru (S.A.M.); tgolihina@mail.ru (T.A.G.); 10 Clinical Diagnostic Centre “Mother and Child Healthcare”, Flotskaya St., 52, 620067 Ekaterinburg, Russia; eozmr-public@mis66.ru

**Keywords:** hyperphenylalaninemia, molecular epidemiology, neonatal screening, phenylalanine hydroxylase, phenylketonuria, *PAH*, *BH4*, tandem mass spectrometry

## Abstract

Since January 2023, the Russian Federation has implemented expanded neonatal screening for 36 hereditary disorders, which has changed the diagnostic algorithm for hyperphenylalaninemia/phenylketonuria (HPA/PKU) by introducing tandem mass spectrometry (MS/MS) on the second day of life, followed by confirmatory biochemical and molecular testing in newborns at risk. We analyzed 1247 newborns aged 5–15 days with elevated phenylalanine levels (≥120 µmol/L) and a phenylalanine to tyrosine ratio of at least 1 detected during the first stage of screening using MS/MS. At the reference center, newborns underwent repeat biochemical testing and stepwise molecular analysis of HPA-associated genes. Two pathogenic variants in HPA-associated genes were identified in 538 newborns, including 534 newborns with biallelic pathogenic variants in *PAH* and 4 with BH4-deficient forms (*PTS*, *QDPR*). The incidence of molecularly confirmed HPA was 1:4518 newborns (95% CI: 1:4152–1:4925). The *PAH* variant spectrum was dominated by p.Arg408Trp (c.1222C>T) (33.4%). Genotype-based analysis indicated that 73 newborns (13.7%) were likely responsive to cofactor therapy, whereas 222 (41.6%) were potentially responsive. These findings define the molecular epidemiology of HPA in Russia and support early genetic stratification for diagnosis and treatment.

## 1. Introduction

Hyperphenylalaninemia (HPA) is a group of autosomal recessive hereditary disorders characterized by elevated blood levels of phenylalanine (Phe). This condition comprises several forms, including phenylketonuria (PKU), BH4-deficient HPA, and non-BH4-deficient HPA [1]. The most common (97–99% of cases) form of HPA is phenylketonuria/hyperphenylalaninemia, which results from impaired activity of phenylalanine hydroxylase (PAH). This impairment is caused by alterations in the nucleotide sequence of the PAH gene, located on chromosome 12 [2]. Elevated concentrations of phenylalanine in the blood and in the brain lead to activation of minor metabolic routes, which results in damage to the central nervous system, global developmental delay, attention deficits, and hyperactivity [3]. The worldwide prevalence of this condition ranges from 0.3 to 38.13 per 100,000 newborns [4], or approximately 1:23,930 individuals (from 1:3139 in Italy to 1:125,000 in Japan) [5,6,7].

Tetrahydrobiopterin (BH4) functions as a cofactor for PAH in the hydroxylation of phenylalanine to tyrosine and also serves as a cofactor for other amino acid hydroxylases, particularly those involved in the biosynthesis of monoamine neurotransmitters [5]. Variants in the *PTS* gene cause BH4 deficiency type A, the most common form (approximately 65% of all BH4-deficient HPA cases in Europe) [6]. BH4-deficient HPA types B, C, and D are associated with variants in the *GCH1*, *QDPR*, and *PCBD1* genes, respectively.

A distinct group of rare causes of hyperphenylalaninemia and/or neurological manifestations resembling dopa-responsive dystonia (levodopa-responsive dystonia) includes disorders affecting the tetrahydrobiopterin (BH4) biosynthesis pathway, such as sepiapterin reductase (*SPR*) deficiency. Notably, GTP cyclohydrolase I deficiency (*GCH1*) may present either as a dopa-responsive movement disorder or as a variant of BH4-associated HPA. Therefore, in the presence of an appropriate phenotype, it is reasonable to consider it within the same group as *SPR*-associated HPA. In addition, a rare form of hyperphenylalaninemia without tetrahydrobiopterin deficiency, caused by pathogenic variants in the *DNAJC12* gene, has also been described [7].

Neonatal screening in the Russian Federation was first introduced in 1993 with the aim of detecting phenylketonuria and congenital hypothyroidism in newborns. Phenylalanine levels were measured on the fourth day of life in dried blood spots on filter paper using a fluorometric method. Since 2023, an expanded neonatal screening (ENS) program has been implemented in the Russian Federation [8]. The introduction of tandem mass spectrometry (MS/MS) for the analysis of amino acids and acylcarnitines in dried blood spots—on the second day of life in full-term newborns and on the seventh day in preterm newborns—enables the determination of phenylalanine levels as well as the calculation of the phenylalanine to tyrosine ratio. Starting in 2023, for the first time in the Russian Federation, the routine expanded neonatal screening protocol officially includes molecular genetic testing for newborns assigned to the risk group. Molecular genetic testing of newborns with biochemically confirmed elevated phenylalanine levels (after a positive result at the first stage of neonatal screening) is currently considered the global gold standard and is routinely performed in the vast majority of developed countries [9].

The clinical classification of hyperphenylalaninemia developed in 1980 and still used in Russian clinical practice distinguishes three biochemical phenotypes of the disease in untreated patients: classical PKU with phenylalanine levels > 1200 µmol/L, moderate HPA (360–1200 µmol/L), and mild HPA (120–360 µmol/L) [1]. Other classification systems further subdivide the disease into four phenotypes, ranging from mild HPA to severe “classical” PKU, characterized by phenylalanine concentrations > 1200 µmol/L [7]. However, there is a growing trend in global clinical practice toward revising these classification criteria. According to the updated 2025 European guidelines, strict adherence to peak untreated Phe values is losing its practical relevance, as modern newborn screening allows for the initiation of therapy before these maximum levels are reached. Consequently, European experts propose a more functional classification, dividing patients with PAH deficiency into three clinical categories: (1) not requiring treatment (Phe < 360 μmol/L); (2) requiring treatment and co-factor (sapropterin) responsive; and (3) requiring treatment and co-factor unresponsive [10]. Despite these differences in classification paradigms, according to Russian clinical guidelines and international standards, dietary therapy, based on replacing natural protein sources with phenylalanine-free products, is applied only when phenylalanine levels exceed 360 µmol/L [11]. In biochemical diagnostics of hyperphenylalaninemia, the phenylalanine to tyrosine ratio in blood is also used. Normally, this ratio is less than 1, whereas values exceeding 3 indicate a high likelihood that HPA is caused by phenylalanine hydroxylase deficiency [12].

The aim of this study was to assess the incidence of hyperphenylalaninemia, the distribution of its different genetic forms, and the spectrum of pathogenic variants in the causative genes in the Russian Federation based on data from the first two years (2023–2024) of the expanded neonatal screening program. Furthermore, we aimed to evaluate the genotype-based predicted responsiveness to BH4 therapy and to analyze the dynamic changes in phenylalanine levels among both affected individuals and heterozygous carriers.

## 2. Results

### 2.1. Epidemiology and PAH Mutation Spectrum

Among the 1247 newborns in the hyperphenylalaninemia risk group whose samples were delivered to the reference center, molecular genetic analysis identified two pathogenic variants in the *PAH* gene in 534 individuals (251 in the first year and 283 in the second year). Notably, at the second stage of biochemical screening (retest), 496 of these cases maintained elevated phenylalanine levels (≥120 µmol/L), whereas in 38 cases, the phenylalanine values had normalized (<120 µmol/L). Two infants were identified with biallelic variants in the *PAH* gene as well as single heterozygous pathogenic variants in other genes (*DNAJC12* in one infant and *GCH1* in the other).

In 4 newborns, two pathogenic variants were detected in genes associated with BH4-deficient forms of HPA: *PTS* (*n* = 2) and *QDPR* (*n* = 2).

A single pathogenic variant in the heterozygous state in the *PAH* gene, after all stages of analysis, was identified in 196 newborns. Among them, 9 also carried heterozygous variants in the *PTS, SPR*, and *GCH1* genes. Similarly, one variant in the *PTS* gene in the heterozygous state was identified in 3 newborns, and one variant in the *PCBD1* gene was identified in 1 case (Table 1).

The full spectrum of variants identified during expanded neonatal screening in Russian newborns is presented in Supplementary Table S1.

**Figure 1:**
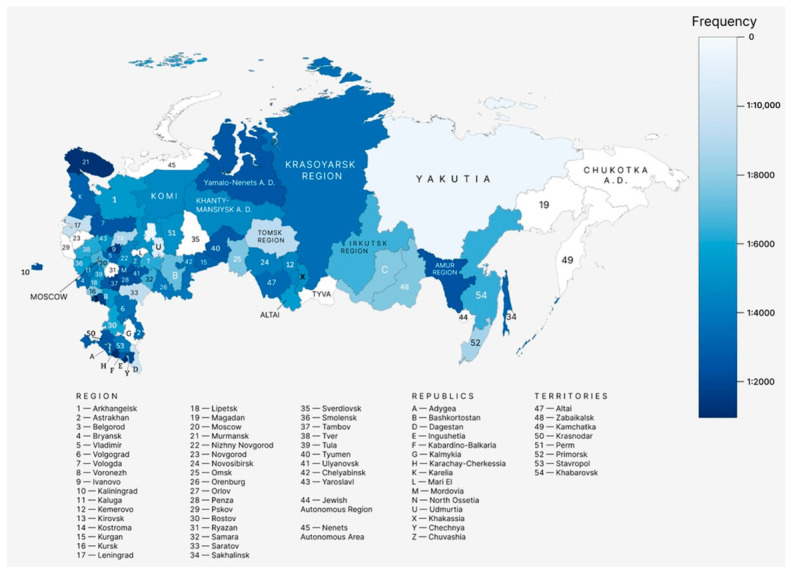
Heat map illustrating the distribution of molecularly confirmed hyperphenylalaninemia incidence across the Russian Federation based on data from 2023 to 2024 (for the Moscow, Sverdlovsk, Kursk, Leningrad, Ulyanovsk, Belgorod, Samara, and Murmansk oblasts, Saint Petersburg, the Udmurt Republic, Ingushetia, and Khakassia, HPA incidence is presented based only on data from the second (2024) year of screening).

The risk group detection rate of hyperphenylalaninemia during primary neonatal screening using tandem mass spectrometry (MS/MS) was estimated to be 1:1934 newborns (*n* = 1257). The incidence of molecularly confirmed HPA in the Russian Federation was 1:4518 (95% CI: 1:4152–1:4925). Initial elevation of phenylalanine levels is often transient, particularly in preterm newborns, due to the immaturity of hepatic enzyme systems as well as the specificities of parenteral nutrition. The incidence of HPA varies from 1:955 in the Republic of North Ossetia–Alania to 1:10,284 newborns in the Udmurt Republic (Figure 1, Supplementary Table S2). The highest incidence of HPA was observed in the North Caucasus Federal District, at 1 case per 2686 newborns. The incidence of PKU (isolated PAH-deficiency, *n* = 534) based on 2023–2024 data was 1:4552 newborns.

All variants identified in the *PAH* gene in Russian newborns have previously been described in the literature as causative of HPA, with the exception of the NM_000277.3:c.353-8T>A variant. This nucleotide variant has not been reported in the control cohort of the Genome Aggregation Database (gnomAD v2.1.1). In silico prediction algorithms assessing its effect on splicing (SpliceAI, Spidex, MMSplice) classify this variant as likely pathogenic. Based on the combined evidence, this nucleotide variant should be classified as a variant of uncertain clinical significance (PM2, PP3, PP4) [13]. This variant was identified in a newborn who also carried a confirmed pathogenic variant in the *PAH* gene, c.916A>G p.(Ile306Val). Consequently, this newborn was attributed to the group with a single pathogenic *PAH* variant and elevated phenylalanine levels (>120 µmol/L) upon retest (Table 1).

Table 2 presents the allele frequencies of *PAH* gene variants detected in 534 newborns with two *PAH* variants. For comparison, the table also includes allele frequencies of *PAH* variants identified in 2579 patients with phenylketonuria who were genotyped during the selective screening program [14].

**Table 2:** The spectrum of *PAH* variants detected during the selective screening program and in 534 newborns with biallelic *PAH* variants identified during the first two years of expanded neonatal screening.

Variant		Residual PAH Activity, %	ENS 2023–2024, Number of Alleles	Allelic Prevalence, % (Total Alleles: 1068)	Selective Screening Prior to 2023, Number of Alleles	Allelic Prevalence, % (Total Alleles: 5158) [14]	Pearson χ^2^ Test, *p* *
p.Arg408Trp	c.1222C>T	5	357	33.4%	2627	50.9%	*X*^2^ = 110.3; *p* < 0.001
p.Ala403Val	c.1208C>T	100	49	4.6%	47	0.9%	*X*^2^ = 78.1; *p* < 0.001
p.Val230Ile	c.688G>A	63	44	4.1%	0	0%	—
p.Ala300Ser	c.898G>T	65	42	3.9%	34	0.7%	*X*^2^ = 85; *p* < 0.001
p.Glu390Gly	c.1169A>G	70	39	3.7%	45	0.9%	*X*^2^ = 50.8; *p* < 0.001
p.Arg261Gln	c.782G>A	47	37	3.5%	273	5.3%	*X*^2^ = 5.2; *p* = 0.024
p.Pro281Leu	c.842C>T	1	30	2.8%	183	3.5%	*X*^2^ = 1.5; *p* = 0.216
IVS10-11G>A	c.1066-11G>A	0	29	2.7%	134	2.6%	*X*^2^ = 0.0; *p* = 0.987
p.Thr380Met	c.1139C>T	28	25	2.3%	0	0%	—
p.Arg261Ter	c.781C>T	39	25	2.3%	72	1.4%	*X*^2^ = 4.1; *p* = 0.043
p.Arg158Gln	c.473G>A	10	19	1.8%	123	2.4%	*X*^2^ = 1.5; *p* = 0.228
p.Arg252Trp	c.754C>T	1	19	1.8%	83	1.7%	*X*^2^ = 0.1; *p* = 0.708
IVS12+1G>A	c.1315+1G>A	0	18	1.7%	159	3.1%	*X*^2^ = 6.4; *p* = 0.012
p.Arg169His	c.506G>A	—	18	1.7%	0	0%	—
p.Arg53His	c.158G>A	—	15	1.4%	0	0%	—
p.Pro211Thr	c.631C>A	72	14	1.3%	0	0%	—
p.Pro119Ser	c.355C>T	—	13	1.2%	0	0%	—
p.Ile306Val	c.916A>G	25	13	1.2%	26	0.5%	*X*^2^ = 7.1; *p* = 0.008
p.Leu48Ser	c.143T>C	47	12	1.1%	51	1.0%	*X*^2^ = 0.2; *p* = 0.702
p.Glu280Lys	c.838G>A	11	12	1.1%	62	1.2%	*X*^2^ = 0.0; *p* = 0.830
p.Tyr414Cys	c.1241A>G	80	11	1.0%	64	1.2%	*X*^2^ = 0.3; *p* = 0.554
p.Val177Met	c.529G>A	—	11	1.0%	0	0%	—
Variants with a prevalence of <1%			216	<1%	1175	<1%	

Note: Variants with residual phenylalanine hydroxylase (PAH) activity greater than 10% are classified as “mild” (indicated in green), whereas those with residual PAH activity below 10% are classified as “severe” (indicated in red). The symbol “—” denotes that residual activity has not been determined. Data are derived from the BIOPKU database (http://www.biopku.org (accessed on 10 October 2025). According to the BIOPKU and PAHvdb approaches, the maximum reported value from published in vitro data was used, with priority given to data obtained in eukaryotic systems (COS-1, HEK-293). * The “χ^2^, *p*” column presents the results of Pearson’s chi-square (χ^2^) test (2 × 2 contingency table) used to compare allele frequencies of a given variant between the two cohorts. To contextualize the observed mutational spectrum in the Russian Federation, we compared it with recently published data from Italy, Northern China and Turkey cohorts (Figure 2).

**Figure 2:**
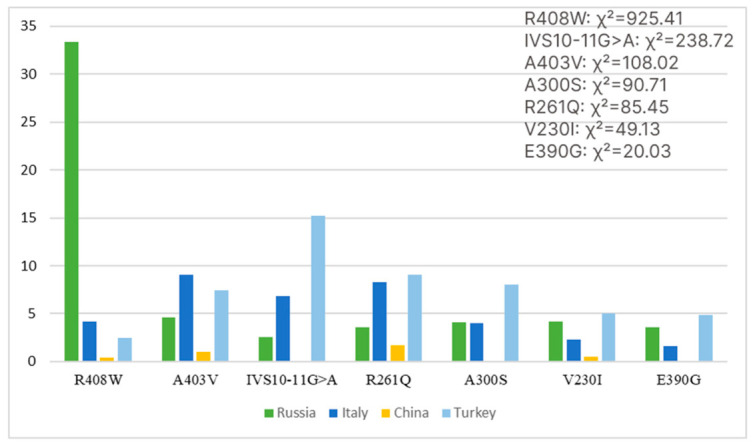
Comparison of the frequencies of the most common *PAH* gene variants in Russia and other countries. Note: Differences in the distribution of common variants between countries were assessed using the χ^2^ test; for all variants, *p* < 0.001. In most cases, degrees of freedom (df) = 3 (four countries), while for p.Glu390Gly, df = 2, as the analysis was performed across three countries, excluding China (the variant was absent in that cohort).

### 2.2. Genetic Profile of BH4-Deficient HPA Forms

Genotypes of 4 newborns with biallelic variants in the BH4-deficient HPA genes are provided in Table 3.

In the *PTS* gene, three previously described variants were identified: c.216T>A (p.Asn72Lys) [15], c.317C>T (p.Thr106Met) [16] and c.108C>G (p.Asn36Lys) [15,17], as well as the variant c.244-1G>T (p.?). The c.244-1G>T (p.?) variant has not been reported in the control cohort of the Genome Aggregation Database (gnomAD v2.1.1). It is located at the canonical acceptor splice site, and in silico tools (MMSplice v2.4.0, SQUIRLS v2.0, SPiP v2.0) predict it to be likely pathogenic, and SpliceAI (v1.3.1) predicts loss of the acceptor splice site.

Additionally, alterations affecting canonical nucleotides at the splice site, such as c.244-2A>G [18] and c.244-2A>T [19], have been described in the literature. Based on this data (criteria PVS1, PM2, PP5), this variant was classified as pathogenic. The applied pathogenicity criteria were based on current guidelines for the interpretation of next-generation sequencing (NGS) data [13].

In the *QDPR* gene, the c.68G>A variant, previously described as pathogenic [20,21], was detected. A novel nucleotide variant, c.538_545delinsTACGGGA, was not reported in the Genome Aggregation Database (gnomAD v2.1.1) control cohort. This variant results in a frameshift and represents a loss-of-function (LoF) change. LoF variants are a known disease mechanism for this gene (32 pathogenic LoF variants have been reported). Based on the combined criteria (PVS1, PM2), this variant was classified as likely pathogenic.

The homozygous state of this variant was confirmed by segregation analysis (Sanger sequencing in a trio format), demonstrating that both parents carried the variant in the heterozygous state.

In the *PCBD1* gene, two variants not previously described as pathogenic, c.184C>T and c.91C>T, were identified in one newborn. These nucleotide variants are reported in the Genome Aggregation Database (gnomAD v2.1.1) control cohort with allele frequencies of 0.00035% and 0.00319%, respectively. In silico prediction algorithms assessing splicing effects (SpliceAI, MMSplice, SQUIRLS, SPiP) classify these variants as neutral. However, comprehensive pathogenicity assessment using meta-predictors MetaSVM and MetaLR classified the variants as pathogenic. Based on the combined evidence (criteria PM2, PP3), these nucleotide variants should be classified as variants of uncertain clinical significance. Due to the lack of convincing evidence supporting their pathogenicity, at this stage, the diagnosis for this newborn cannot be considered molecularly confirmed.

### 2.3. Phenylalanine Dynamics During the Neonatal Period

**Figure 3:**
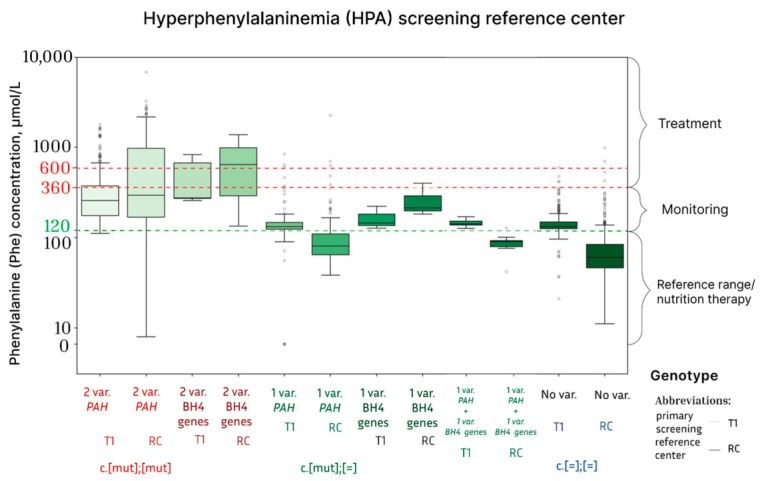
Distribution of phenylalanine (Phe, µmol/L) concentrations in newborns according to data from regional centers (first stage of expanded neonatal screening) and the reference center.

A detailed analysis of phenylalanine levels was conducted at primary and repeat blood sampling in newborns according to genotype categories: (1) two pathogenic variants in the *PAH* gene; (2) two pathogenic variants in genes associated with BH4-deficient HPA; (3) one pathogenic *PAH* variant; (4) one pathogenic variant in genes associated with BH4-deficient HPA; (5) multiple heterozygous variants in different HPA-associated genes; and (6) no sequence variants identified in HPA-associated genes (Figure 3).

Median phenylalanine levels were analyzed in groups of newborns with different genotypes during primary screening (performed on the second day of life in full-term newborns and on the seventh day in preterm newborns) and during repeat analysis of blood samples collected on days 5–15 of life (Table 4).

According to the data presented in Figure 3 and Table 4, statistically significant differences in median phenylalanine levels between primary screening and confirmatory retesting were observed in most of the studied groups. Exceptions included newborns with variants in genes associated with BH4-deficient forms of hyperphenylalaninemia (both in the presence of two pathogenic variants and in the heterozygous state).

### 2.4. Spectrum of PAH Variants in Heterozygous Carriers

**Figure 4:**
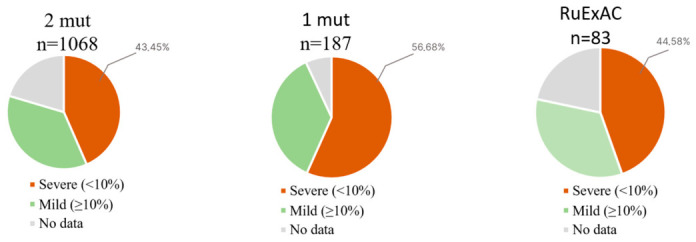
Spectrum of PAH variants based on residual PAH activity in cohorts with various genotypes.

A comparative analysis of the spectra of pathogenic variants was performed among newborns with biallelic *PAH* variants (*n* = 1068 chromosomes), heterozygous carriers included in the risk group (*n* = 187 chromosomes), and randomly selected carriers from the Russian exome aggregation database (RuExAC) comprising patients with various hereditary diseases (*n* = 83 chromosomes) (Figure 4).

When comparing the distribution of severe (<10% residual PAH activity) and mild (≥10% residual PAH activity) variants (excluding variants lacking activity data), no statistically significant differences were observed among the three groups: newborns with two variants, carriers from the risk group, and incidental carriers (Pearson’s χ^2^ test = 2.386, *p* = 0.303). Although a slight tendency toward a higher proportion of severe variants was noted in the risk group (56.7% vs. 43.5% in the group with two variants), these differences did not reach statistical significance.

### 2.5. Genotype-Based Prediction of BH4 Responsiveness

**Figure 5:**
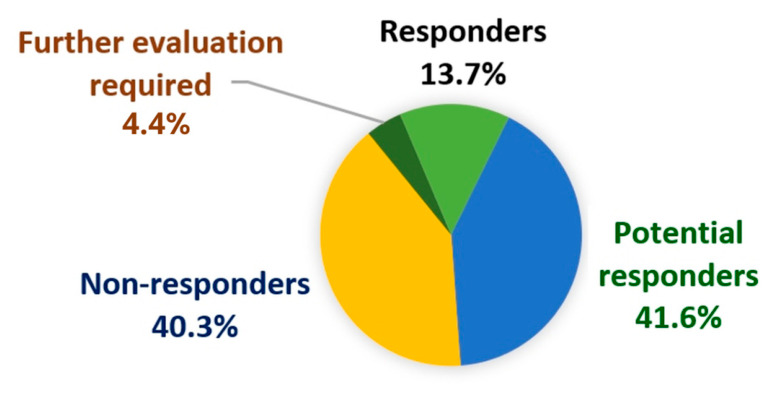
Proportions of potential responders and non-responders to sapropterin (BH4) therapy.

During molecular genetic analysis, 534 newborns with two pathogenic variants in the *PAH* gene were identified (Table 1). Prediction of therapeutic response to the phenylalanine hydroxylase cofactor was based on analysis of residual PAH activity determined for the corresponding genotypes according to the BIOPKU database. The BIOPKU database contains results of in vitro expression studies of altered PAH protein variants, including their residual enzymatic activity relative to the wild-type enzyme. Patients with two “severe” variants (residual PAH activity < 10%) in the *PAH* gene do not respond to sapropterin (BH4) therapy, as such low overall enzymatic activity precludes a sufficient stabilizing effect of exogenous BH4 on the altered protein conformation. Patients carrying at least one “mild” variant (residual activity ≥10%) were considered potentially responsive to therapy; however, for these patients, loading tests are required to guide treatment decisions. Studies have shown that a minimum residual PAH activity of at least 12.5–15.5% is required to achieve a response to BH4 [22]. As a result, 73 newborns (13.7%) identified through screening in 2023–2024 were classified as responders to cofactor therapy based on the residual PAH activity characteristic of their genotypes (two “mild” variants), 222 newborns (41.6%) were considered potential responders, and 215 individuals (40.3%) had genotypes predicting non-responsiveness to therapy. In 24 newborns (4.4%), variants with unknown residual PAH activity (not annotated in BIOPKU) were identified (Figure 5).

Prediction of therapeutic response to the BH4 cofactor was assessed. The predictive response rates in our study cohort are summarized in Table 5.

## 3. Discussion

### 3.1. Epidemiology and Mutation Spectrum of PAH Deficiency

The prevalence of phenylketonuria (PKU) observed in our study (1:4552) indicates that the Russian Federation is one of the countries with a high detection rate of this disorder. In this epidemiological context, we use the term ‘PKU’ to encompass the entire spectrum of PAH deficiency, including mild HPA cases, based on our molecular genetic findings (biallelic *PAH* variants). 

While the national average incidence has increased compared to previous estimates, this trend is particularly evident when analyzing specific regional shifts (Figure 1, Supplementary Table S2). In a previous 2021 study conducted in the Russian Federation, the highest incidence of PKU was reported in the Republic of Mordovia (1:4000 newborns), while the lowest was observed in the Arkhangelsk Oblast (1:17,000 newborns) [23]. However, according to the present data, the incidence in these regions has changed to 1:2031 and 1:6514, respectively, shifting their positions within the national ranking. Such pronounced changes in these regions can be explained by several factors. Firstly, the implementation of expanded neonatal screening from 2023 and the transition to MS/MS in primary screening have led to the inclusion of cases of mild HPA, which may previously have remained undiagnosed. This has naturally increased the observed incidence of the disease. Secondly, the statistical phenomenon of small numbers must be considered: in regions with low birth rates (such as Mordovia and the Arkhangelsk Oblast), the occurrence of even 1–2 additional cases per year can lead to substantial fluctuations in calculated incidence rates. Therefore, the observed changes in disease incidence in these regions are not attributable to true shifts in the genetic structure over the past three years, but rather to the introduction of more sensitive diagnostic criteria within expanded neonatal screening, as well as statistical variability in small cohort sizes.

This shift in incidence is closely tied to changes in the observed mutation spectrum (Table 2). The most common variant in Russia is p.Arg408Trp, accounting for 33.4% of the total number of alleles in the cohort of newborns with two identified pathogenic variants. However, this proportion is lower than that reported in previous Russian studies (50.9%) (Table 2) [14]. A similar dynamic is observed for other pathogenic variants with zero or extremely low residual enzyme activity. For example, the frequency of the IVS12+1G>A variant decreased from 3.1% to 1.7% (χ^2^ = 6.4; *p* = 0.012), and that of the p.Arg261Gln missense variant decreased from 5.3% to 3.5% (χ^2^ = 5.2; *p* = 0.024). Particularly noteworthy are variants that were previously among the more common but demonstrated a marked decline in frequency according to ENS data. For instance, the p.Asp222* (c.664_665delGA) variant, which ranked 8th in frequency in the selective screening cohort (1.4%, 72 alleles), accounted for only 0.9% (10 alleles) in our cohort. A similar decrease was observed for the IVS4+5G>T variant, whose frequency declined from 1.2% to 0.7%. In contrast, the frequency of several “mild” variants associated with moderate and mild forms of hyperphenylalaninemia increased substantially in the ENS cohort. Variants with high residual enzyme activity, p.Ala403Val, p.Val230Ile, and p.Ala300Ser, which were previously observed at frequencies below 1%, became leading variants in the ENS dataset, with frequencies of 4.6%, 4.1%, and 3.9%, respectively. The use of a comprehensive approach within expanded neonatal screening has led to improved detection of newborns with mild HPA forms, which previously may have produced borderline results and remained undiagnosed. Detecting these mild cases is of significant clinical utility. Although these infants may not require immediate dietary restriction, early diagnosis allows for regular monitoring to prevent future phenylalanine spikes and their potential detrimental effects on neurodevelopment, and it is crucial for anticipating the need for strict dietary management during future pregnancies to prevent maternal phenylketonuria syndrome.

To contextualize the high incidence of PKU observed in our cohort within the global landscape, it is important to note that the highest PKU prevalences are reported in Europe and certain countries of the Middle East. For example, higher prevalences have been reported in Italy (1:3139) [24] and Ireland (1:4078) [25] compared to Jordan (1:5263) [26]. In all of these countries, national screening programs are based on tandem mass spectrometry (MS/MS).

A PKU prevalence comparable to that observed in Russia has also been reported in Turkey (1:4500) [27]. However, direct comparison is methodologically limited, as primary mass screening in Turkey is conducted using a fluorometric immunoassay, which has lower sensitivity than tandem mass spectrometry [28]. Consequently, the true incidence of the disease in that country may be even higher than reported.

In other studies, the highest frequency of the p.Arg408Trp variant has been reported in Lithuania (73.5%) [29]. Among European patients, p.Arg408Trp also predominates (mean allele frequency, AF = 63.7%), followed by IVS10-11G>A (AF = 11%) and p.Arg261Gln (AF = 11%). In Eastern Europe, the p.Arg408Trp variant is also the most common (54.6%), and a substantial proportion of cases is associated with the homozygous genotype p.Arg408Trp/p.Arg408Trp (genotype frequency, GF = 32.7%). A similar tendency is observed in Central Europe (AF = 44.4%, GF = 23.8%) [30]. International reviews indicate that, for some countries, published data lack standardization in both the method of phenylalanine measurement and the patient’s age at testing, which further limits the interpretation of inter-country differences.

The second most frequent variant in Russia is p.Ala403Val (4.6%). In other countries, its prevalence is higher, for example, 13.6% in Slovenia and 10.1% in the Czech Republic. The third most common variant is p.Val230Ile (4.1%). Globally, p.Val230Ile is considered a rare variant, with its highest frequency reported in the Middle East, particularly in Iran (allele frequency 2.5%) [31], compared to 0.22% in Portugal [32]. The fourth most common variant, p.Ala300Ser (3.9%), has comparable frequencies in Iran (3.8%) and Spain (3.7%). The p.Glu390Gly variant was observed in Russia at a frequency of 3.7%, whereas in Slovenia it accounts for 22.7%, in Croatia 14.3%, and in Austria 5.6% [30].

These population-specific differences become even more apparent when performing a direct comparison of the most common PAH variants against large, recently published international cohorts from Italy (*n* = 758), northern China (*n* = 655), and Turkey (*n* = 399 patients, 735 alleles) (Figure 2). In Italy, the most frequent variant was p.Ala403Val (9.8%; 149 alleles), followed by p.Arg261Gln (8.29%; 126) and IVS10-11G>A (6.78%; 103), whereas p.Arg408Trp was less common (4.21%; 64) [33]. In northern China, the frequencies of these variants were low: p.Val230Ile 0.5%, p.Arg261Gln 1.7%, p.Ala300Ser 0.1%, p.Arg408Trp 0.4%, p.Ala403Val 1.0%, and IVS10-11G>A 0.1% [34]. In Turkey, the most frequent variant was IVS10-11G>A (15.2%; 112 alleles), followed by p.Arg261Gln (9.1%; 67), p.Ala300Ser (8.0%; 59), p.Ala403Val (7.5%; 55), p.Val230Ile (5.0%; 37), and p.Glu390Gly (4.9%; 36), while p.Arg408Trp was less common (2.4%; 18) [35].

It should be noted that in the analyzed international cohorts, primary screening was conducted using different methodologies (primary screening for phenylketonuria in Turkey was based on a fluorometric method [28], whereas the screening methodology varied across regions in Italy [33] and China [36], resulting in mixed approaches), in contrast to the exclusive use of tandem mass spectrometry in our cohort. Because MS/MS allows for precise quantification of both phenylalanine levels and the phenylalanine to tyrosine ratio, it provides a higher diagnostic yield for mild hyperphenylalaninemia variants. Consequently, the true overall incidence of the disease in countries relying on older or mixed protocols might be somewhat underestimated compared to our findings. Nevertheless, despite these methodological differences impacting absolute incidence rates, the comparison of allele frequencies between countries remains valid. The allelic spectrum of pathogenic variants in the *PAH* gene varies substantially between countries, as confirmed by statistically significant differences in the distribution of major variants.

### 3.2. Prevalence and Genetic Spectrum of BH4-Deficient Forms

Returning to the composition of the Russian HPA cohort, it is important to highlight that within the framework of the expanded neonatal screening program in the Russian Federation, a large cohort of newborns with a confirmed diagnosis of HPA was characterized. Among 538 molecularly confirmed cases, 534 were caused by pathogenic variants in the PAH gene (99.3%), while 4 cases represented BH4-deficient forms (0.7%).

The observed proportion of BH4-deficient forms is consistent with data from a large retrospective study by P. Gundorova et al. (2021), in which tetrahydrobiopterin deficiency accounted for 0.87% of all HPA cases in the Russian Federation (30 out of 3452 patients) [15]. However, the authors noted that this figure is likely overestimated due to selection bias: diagnostically complex cases were more frequently referred to the federal center, whereas patients with common PAH gene mutations were often diagnosed at the regional level. Taking this factor into account, the true prevalence of BH4 deficiency in the Russian Federation was estimated by the authors at approximately 0.4%, a value previously demonstrated in a smaller but more representative cohort by I.A. Kuznetsova et al. [23,37]. PTS deficiency (6-pyruvoyl-tetrahydropterin synthase deficiency) is the most common BH4-deficient form of HPA worldwide [38]. In our relatively small cohort of newborns from the Russian Federation, mutations in the *PTS* and *QDPR* genes were observed with equal frequency.

Despite methodological limitations in estimating the overall prevalence, the study by P. Gundorova et al. remains the most comprehensive source of data on the allelic diversity of BH4-dependent forms in the Russian Federation. The comparison between the variant spectrum identified in our study and previously published data was of particular interest. The mutation spectrum of the PTS gene (Table 3) in our cohort demonstrated a high degree of concordance with data from 2021. Thus, one newborn was found to carry a compound heterozygous genotype for the missense variants, c.216T>A (p.Asn72Lys) and c.317C>T (p.Thr106Met). These two variants constitute the major mutation pool of the PTS gene in the Russian Federation, accounting for 20% and 32% of alleles, respectively. Such a frequently observed compound heterozygous state suggests a relatively high carrier frequency of these alleles in the Russian Federation. This assumption is supported by data from the Russian exome aggregation database (RuExAC), according to which the c.317C>T variant occurs with an allele frequency of 0.07% (4 alleles in 5820 chromosomes). In the second newborn, the c.108C>G (p.Asn36Lys) missense variant was detected in a compound heterozygous state with the c.244-1G>T variant. While the p.Asn36Lys variant had previously been reported in a Russian patient, the identification of the c.244-1G>T variant contributes to the expansion of the mutation spectrum previously described for the Russian Federation. Subsequent segregation analysis confirmed that both the c.108C>G and the novel c.244-1G>T variants were inherited in trans from the heterozygous parents.

In contrast to the *PTS* gene, for which a clear accumulation of major variants is observed, the *QDPR* gene is characterized by pronounced allelic heterogeneity in the Russian patient cohort. In the study by Gundorova et al. [15], five different *QDPR* mutations were described in four Russian patients; none of these variants were identified in our cohort. It is noteworthy that both newborns examined in our study were homozygous for the identified rare variants (Table 3), and these cases were registered in specific territorial and ethnic groups (one child from the Republic of Buryatia and the other from Altai Krai). Moreover, the detected *QDPR* variants are absent from the RuExAC database. A similar pattern was observed in the study by Gundorova et al., where 3 out of 4 patients with *QDPR* deficiency also exhibited a homozygous genotype [15]. This suggests that the overall carrier frequency of *QDPR* gene mutations in the Russian Federation is relatively low, while disease manifestation in the homozygous state is strongly local and ethnically based.

Beyond genotype prevalence, it is critical to evaluate the biochemical phenotype of these rare cases, as it directly dictates clinical management. In newborns with BH4-deficient forms, the mean phenylalanine level was 687 µmol/L, indicating pronounced hyperphenylalaninemia with a high risk of affecting the central nervous system. Because of the elevated neurological risk, pharmacological therapy for BH4-deficient HPA forms is pathogenetically targeted and is initiated immediately after confirming diagnosis without the need for loading tests [1].

### 3.3. Dynamics of Phenylalanine Levels at Primary and Repeat Screening

When evaluating the clinical implications of our findings, the largest range in Phe levels was observed in the group of newborns with two *PAH* variants during repeat testing (Figure 3, Table 4). This may be attributed to differences in genotypes, the interval between birth and repeat blood sampling, and the fact that for some infants, dietary therapy has been prescribed by the time of the second test.

In 58.6% of newborns with two pathogenic *PAH* variants, repeat screening revealed Phe levels in the range of 120–360 µmol/L (overall range: 7–3227 µmol/L; mean at primary screening = 318 µmol/L; mean at retest = 619 µmol/L) (Table 4). According to Russian clinical guidelines, such patients require continuous Phe monitoring with measurement of fasting Phe levels: during the first 3 months of life, once weekly (until stable values are achieved), then once every 10 days; from 1 to 6 years of age, at least 1–2 times per month. In European countries, recommended monitoring is more frequent: up to 1 year of age—once per week; from 1 to 12 years—once every 2 weeks; over 12 years—once per month; during preconception—once per week; and during pregnancy—twice per week [39].

A particularly important subset for clinical management decisions is those whose values normalized. It is worth noting that in 7.1% of newborns with two pathogenic *PAH* variants, repeat testing showed Phe levels within the normal range (<120 µmol/L). According to Russian guidelines, clinical management and treatment decisions are based on the actual blood Phe concentration rather than solely on the presence of pathogenic variants in HPA-associated genes. Although dietary therapy is not prescribed for these patients, Phe levels should be monitored during the first year of life as follows: once per week during the first month and at least once per month from the second month to one year.

Genotype analysis in this subgroup revealed a marked predominance of alleles associated with high residual enzyme activity in vitro. Thus, in approximately half of these newborns (19 out of 38), at least one “mild” variant with residual activity greater than 10%, such as p.Ala300Ser, p.Arg261Gln, or p.Ala403Val, was present in a compound heterozygous state. At the same time, in 4 children, low Phe levels were observed despite homozygosity for the “severe” variant p.Arg408Trp, which may be explained by insufficient protein intake during the first days of life.

Particular attention should be paid to the group of newborns (*n* = 38) in whom, despite the identification of two pathogenic variants in the *PAH* gene, Phe levels remained within the normal range (<120 µmol/L) upon repeat testing (Table 1). This fact raises important questions regarding the sensitivity and clinical utility of the new screening algorithm compared to previous protocols. Previously, prior to 2023, the recorded incidence of HPA in Russia was approximately 1:7000 newborns [40]. In the present study, the incidence of molecularly confirmed HPA was 1:4518. Notably, even if the cohort of asymptomatic newborns with low Phe levels (<120 µmol/L) at the time of screening is excluded from the analysis, the incidence remains high at 1:4881. This demonstrates that previous screening approaches failed to detect not only patients with transiently normal Phe levels but also a substantial proportion of children with true HPA.

Among newborns with one heterozygous pathogenic *PAH* variant, 80.2% had normal Phe levels (<120 µmol/L) upon retesting (mean at primary screening = 151 µmol/L; mean at retest = 109 µmol/L). For newborns in whom a second variant was not identified but Phe levels remained > 120 µmol/L, clinical management depends on the current phenylalanine concentration. According to clinical guidelines, specialized dietary therapy is initiated at Phe levels ≥ 360 µmol/L, while patients with Phe values between 120 and 360 µmol/L warrant dynamic follow-up (from 2 to 12 months of age: once per month) until a definitive diagnosis is established.

In newborns without identified pathogenic variants in HPA-associated genes, a wide range of Phe concentrations was observed at primary screening (21–992 µmol/L). In 90.4% of cases, hyperphenylalaninemia was transient, with normalization of Phe levels upon repeat testing (mean at primary screening = 145 µmol/L; mean at retest = 76 µmol/L).

In the group of newborns with two variants in genes associated with BH4-deficient forms of HPA, phenylalanine levels remained elevated both at primary screening and upon repeat analysis (range: 135–1374 µmol/L; mean at primary screening = 408 µmol/L; mean at retest = 787 µmol/L). Despite the absence of statistically significant differences between primary and repeat measurements, a high proportion (75%) of cases with Phe levels ≥ 360 µmol/L necessitates mandatory initiation of therapy. In such cases, identification of the molecular genetic cause is of critical importance: accurate verification of a BH4-deficient form enables timely initiation of targeted pharmacological treatment, thereby preventing irreversible damage to the central nervous system.

An increase in Phe levels, although not statistically significant given the small sample size, was also observed in newborns with a single pathogenic variant in the *PTS* gene (*n* = 3) and in the *PCBD1* gene (*n* = 1), which are associated with BH4-deficient HPA types A and D, respectively. The *PCBD1* gene encodes pterin-4α-carbinolamine dehydratase, defects of which are known to cause transient neonatal hyperphenylalaninemia [41]. Metabolic instability in heterozygous carriers of *PCBD1* variants may explain the dynamic increase in Phe levels during the first year of life. For the *PTS* gene, both gross deletions and deep intronic variants leading to the formation of cryptic splice sites have been described [42]. However, the gene panel used in this study covers only intronic regions adjacent to exons. The combination of biochemical findings and characteristics of the mutation spectrum in the PTS gene necessitates continued analysis of the genetic causes of HPA in newborns with a single heterozygous PTS variant and dynamically elevated Phe levels.

In contrast, newborns with a single heterozygous variant in the *PAH* gene or no identified variants exhibited a dynamic decrease in median Phe levels, highlighting the complexity of biochemical processes in newborns and the possibility of false-positive results at the first stage of screening.

### 3.4. Potential Mechanisms of Transient Hyperphenylalaninemia in Heterozygous Carriers

According to the results of expanded neonatal screening, 538 probands have two identified pathogenic variants in HPA-associated genes (534 in the *PAH* gene and 4 in genes associated with BH4-deficient forms). Based on the total number of newborns screened in the Russian Federation in 2023–2024, the incidence of newborns with HPA carrying two pathogenic variants confirming the diagnosis at the molecular level was 1:4518, while the incidence of *PAH*-associated HPA was 1:4552. Thus, at least 1 in 34 individuals in Russia is a carrier of a pathogenic variant in the *PAH* gene. The expected number of heterozygous carriers randomly included in the risk group would be 37. However, 196 heterozygous carriers of pathogenic *PAH* variants were actually identified in the risk group, exceeding the expected value by more than fivefold. This finding suggests a non-random enrichment of carriers among newborns with elevated phenylalanine levels at birth.

To explain this observation, the following hypotheses were proposed:

1. In heterozygous carriers, phenylalanine hydroxylase activity is reduced to approximately 50%, which, under neonatal conditions (functional immaturity of the liver, altered protein metabolism, and possible perinatal stress factors), may be insufficient for normal phenylalanine metabolism. Consequently, some carriers may exhibit transient or borderline increase in Phe levels and be included in the risk group at the first stage of screening.
2. The presence of a second pathogenic variant in heterozygous individuals that remains undetected because of limitations of the screening methods used.
3. Elevated phenylalanine levels at birth may result from combined carrier status in both the mother and the fetus.
4. A cumulative effect of adverse genetic and non-genetic factors in the perinatal period, the presence of modifying factors, including variants in other genes involved in BH4 metabolism.

Regarding the dynamic changes in phenylalanine levels between primary and repeat screening (Figure 3, Table 4), the lack of statistically significant differences in certain groups, such as newborns with BH4-deficient forms, is attributable to their small size and genetic heterogeneity. Since not all heterozygous carriers are included in the HPA risk group at the first stage of screening, it can be hypothesized that this cohort is primarily enriched with carriers of functionally “severe” pathogenic variants. A substantial reduction in the overall residual phenylalanine hydroxylase (PAH) activity, contributed by the affected allele, increases the likelihood that a heterozygous newborn will fall into the “gray zone” of phenylalanine levels during the neonatal period and, consequently, be referred for second-stage evaluation. This assumption aligns with our comparative analysis (Figure 4), which revealed a notable tendency toward a higher proportion of severe variants in the risk group (56.7%) compared to the biallelic newborn cohort (43.5%). Although this difference did not reach statistical significance in our current sample size, the observed trend suggests that severe alleles may exert a stronger negative effect on overall enzyme activity even in the heterozygous state. Nevertheless, while the enrichment of severe variants is evident, heterozygosity for a single “severe” PAH variant cannot be considered the sole factor explaining the high detection rate of carriers in the screening program. This suggests that other compounding variables likely act synergistically to cause transient hyperphenylalaninemia.

In addition to gross deletions, deep intronic variants have also been described in the PAH gene, including in Russian patients with PKU [43]. Therefore, it cannot be excluded that a subset of carriers, particularly those who retain elevated Phe levels at the second stage of screening, harbor a second pathogenic variant located in unexamined regions, necessitating further investigation using genome-wide methods. However, according to previous studies [23], the proportion of patients with such variants in the overall cohort does not exceed 1%. Thus, the expected number of newborns carrying variants undetectable by the methods used is approximately 3–7 individuals. Consequently, the presence of a second undetected variant cannot account for all cases of heterozygotes included in the risk group.

An elevated Phe level at birth may also result from the combined carrier status of both the mother and the fetus. The key mechanism underlying increased fetal phenylalanine levels in the context of maternal carrier status is the active transport of phenylalanine across the placenta. In cases where the mother carries the most common severe variant in the Russian Federation, p.Arg408Trp, even a moderate increase in phenylalanine levels may lead to transient elevation of Phe in the newborn during primary screening. Among 83 PAH gene pathogenic variant carriers in the RuExAC database, the p.Arg408Trp variant was identified in 32 individuals (out of 2910 individuals), corresponding to an observed carrier frequency of 1.1% (approximately 1 in 91 individuals).

The probability of transmitting carrier status to the fetus from a carrier mother is 50%. The probability of the combination “carrier mother + carrier fetus” for the p.Arg408Trp (c.1222C>T) variant is therefore 0.011 × 0.5 = 0.0055 (0.55%). Thus, in 2023–2024, more than 13,000 children carrying the p.Arg408Trp (c.1222C>T) variant in the heterozygous state inherited from the mother should have passed through the neonatal screening program. This value exceeds by more than 60-fold the total number of heterozygous PAH gene carriers identified within the risk group. Therefore, the combination of maternal and fetal carrier status alone is insufficient to explain elevated Phe levels in newborns.

An increase in Phe levels after birth could also be explained by the cumulative effect of unfavorable genetic and non-genetic factors during the perinatal period, as well as by the presence of modifying factors, including heterozygous variants in other genes involved in tetrahydrobiopterin (BH4) metabolism. In 9 newborns with elevated Phe levels (>120 μmol/L) at primary testing, *PAH* gene variants co-occurred with variants in other genes associated with tetrahydrobiopterin metabolism. During repeat MS/MS testing, Phe levels in 8 out of 9 children were within the reference range, indicating the transient nature of hyperphenylalaninemia in carriers of heterozygous variants in more than one gene associated with HPA.

Thus, the main cause of the increased detection of heterozygous carriers in biochemical screening could not be determined; most likely, the underlying reasons are individual in each specific case. Zhang W. et al. demonstrated that heterozygous carrier status may be associated with a statistically significant shift in relevant biochemical markers (e.g., elevated Phe levels in carriers of *PAH* variants), thereby increasing the likelihood that such individuals fall into borderline cutoff zones during neonatal screening [44]. The detection of heterozygous carriers during biochemical neonatal screening has also been reported for other inborn errors of metabolism. For example, in screening for MCAD deficiency, elevated C8 levels in a subset of “false-positive” newborns are associated with heterozygous carriage of the common ACADM variant (c.985A>G) [45]. Similarly, literature data indicate that heterozygous carriage of pathogenic *PAH* variants is frequently accompanied by a moderate elevation in phenylalanine levels. According to a recent comprehensive review by Khan et al. [46], genetic carriers exhibit higher phenylalanine concentrations, lower tyrosine concentrations, and an elevated phenylalanine to tyrosine ratio compared to healthy non-carriers, reflecting a partial reduction in phenylalanine hydroxylase activity. This is further supported by classic metabolic studies, such as the work by Verduci et al. [47], which demonstrated that *PAH* heterozygotes display altered phenylalanine and tyrosine metabolism both fasting and after a protein load. Specifically, carriers showed a blunted increase in tyrosine levels upon transitioning from a fasting to a post-load state, highlighting a diminished capacity for phenylalanine hydroxylation. Interestingly, while baseline phenylalanine differences between carriers of “severe” versus “mild” variants were less pronounced, alterations in tyrosine dynamics proved to be a highly sensitive marker of the genotype. Taken together, these metabolic shifts clarify why a substantial number of healthy *PAH* carriers present with transient or borderline hyperphenylalaninemia, causing them to fall into the risk group during the highly sensitive primary MS/MS screening. Therefore, the high proportion of heterozygous variants among children referred to the second stage of screening is expected and underscores the importance of retesting and dynamic follow-up until the diagnosis is definitively excluded.

### 3.5. Prediction of BH4 Responsiveness

Based on the results from the first two years of combining biochemical neonatal screening for HPA using MS/MS with molecular genetic analysis, the spectrum of *PAH* gene variants identified in children with HPA has shifted compared with data obtained from molecular genetic screening prior to 2023 toward a predominance of variants with high residual PAH activity (Table 2). These findings are consistent with recent international observations. For example, in Sweden, a significant increase in the proportion of patients with mild hyperphenylalaninemia has been reported since 1990, attributable to the implementation of more sensitive screening methods [48]. Despite the growing proportion of “mild” variants, p.Arg408Trp remains the most common pathogenic variant in the Russian Federation (allele frequency among all genetically confirmed PAH-related HPA cases is 33.4%).

One of the major challenges in the management of PKU is predicting disease severity and progression based solely on genetic data. According to the BioPKU project, genotype data reliably predict phenotype but have limited accuracy in forecasting responsiveness to BH4 [49]. In cases where a patient carries two mild *PAH* variants, residual enzyme activity may reach 25–75% of normal, which explains moderately elevated phenylalanine levels during the neonatal period [14]. Meanwhile, the less severe of the two variants determines the clinical manifestation of the disease, which explains why newborns carrying one “severe” variant in combination with a “mild” variant may exhibit phenylalanine levels comparable to those observed in patients with two “mild” variants [50,51]. Clinical guidelines define a threshold value of 360 μmol/L as the critical point for initiating dietary therapy, corresponding to the boundary between observation and active treatment. Adaptation to protein load in newborns with “mild” variants occurs gradually. During the first months of life, prior to the introduction of complementary foods with high protein content, phenylalanine levels may remain within the physiological range. A critical period arises with the introduction of meat products, when protein intake increases from 1.5 to 2.0 g/kg body weight during breastfeeding to 3–4 g/kg with complementary feeding [52].

To evaluate whether the shift in the variant spectrum observed in our ENS cohort is reflected in the predicted therapeutic response, we compared these findings with previously published data from earlier Russian studies [14]. According to previous studies involving genotyping of 2679 PKU patients, the following results were obtained: 56.0% of probands (1444 patients) carried “severe” variants in homozygous or compound heterozygous states and comprised a cohort of non-responders, while at least one “mild” variant was identified in 20.2% of probands (520 patients). Overall, 55.1% of newborns carry at least one “mild” variant in their genotype, making them potential candidates for phenylalanine hydroxylase cofactor therapy (Figure 5).

As detailed in Table 5, in international cohorts, the proportion of BH4 responders was reported as follows: Italy—44% (*n* = 46) [53]; Japan—29.1% (*n* = 203) [54]; China—25.43% (*n* = 346) [55]; Serbia—51% (*n* = 61) [56]; and Spain—34.45% (102 of 296 tested individuals) [57], based on the assumption that the presence of a specific variant in at least one allele of the PAH gene is sufficient for a positive response to BH4. A study conducted in the Netherlands demonstrated a 61% response rate using a 48-h loading test in 177 patients (genotype data were available for 120 individuals) [51]. The proportion of potential responders to cofactor therapy in Russia does not differ significantly from that observed in other European countries, including the Netherlands, Italy, and Serbia, and is significantly higher than in Asian countries such as Japan and China and in other countries, such as Spain.

Thus, the presented data indicate heterogeneity among the detected cases of hyperphenylalaninemia: the cohort includes both “mild” variants and clinically more severe forms, necessitating a systematic therapeutic approach and long-term patient monitoring in order to optimize treatment outcomes.

## 4. Materials and Methods

In the Russian Federation, expanded neonatal screening (ENS) is implemented as a nationwide public health program and is universally offered to all liveborn infants. Participation in ENS is voluntary, and screening is performed only in the presence of written informed consent signed by a parent. Parents retain the legal right to refuse medical interventions, including neonatal screening, and may decline participation in the NBS program by providing a written informed refusal (a handwritten statement of voluntary renunciation) bearing their signatures, as provided by Federal Law No. 323-FZ “On the fundamentals of health protection of citizens in the Russian Federation” and relevant national regulations. The implementation of ENS, including sample collection and the routine application of subsequent biochemical and molecular genetic testing, is regulated by a number of normative legal acts. The key document governing ENS implementation is Order of the Ministry of Health of the Russian Federation No. 274n, dated 21 April 2022, “On approval of the procedure for providing medical care to patients with congenital and/or hereditary diseases”. Consequently, the present study represents a retrospective analysis of the outcomes generated by this officially established clinical workflow, in which informed parental consent for participation in the screening program had been obtained for all analyzed cases.

In 2023 and 2024, 2,430,554 newborns underwent expanded neonatal screening (coverage rate: 97.3%). Based on the results of the primary biochemical tandem mass spectrometry (MS/MS) test on dried blood spots collected on the second day of life (24–48 h) in full-term newborns and on the seventh day (144–168 h) in preterm newborns (defined as born before 37 weeks of gestation), a risk group for hyperphenylalaninemia was formed, comprising 1257 newborns (573 in the first year and 684 in the second year of screening).

The inclusion criteria for the HPA risk group were: elevated phenylalanine (Phe) levels ≥120 µmol/L and a phenylalanine to tyrosine ratio >1. It should be noted that 20 newborns were included in the risk group and referred to the reference center despite having primary phenylalanine levels below the standard threshold (≤120 µmol/L). This occurred for two reasons: either the borderline phenylalanine levels were accompanied by an elevated phenylalanine to tyrosine ratio (>1), or the primary screening showed normal phenylalanine but deviations in other amino acids/acylcarnitines, while the subsequent confirmatory MS/MS retest at the reference center registered a secondary elevation in phenylalanine. Consequently, these newborns underwent the complete HPA molecular genetic testing protocol, which identified individuals with two pathogenic *PAH* variants, heterozygous carriers and cases with no identified variants.

Clinical geneticists working in the interregional NBS centers are responsible for reviewing screening results and communicating positive findings to the parents and/or the attending pediatricians at the local hospital within 24 h after a positive primary screening result is obtained. During this communication, they also outline the recommended clinical management strategy, including the follow-up schedule and, when appropriate, initiation of a low-phenylalanine diet or other pathogenetically targeted measures.

All screen-positive cases are referred to the national reference center for confirmatory testing. Newborns with a positive result at the first stage of MS/MS-based screening are recalled for repeat sampling at 5–15 days of life. During this recall visit, a new dried blood spot sample is obtained for confirmatory biochemical testing by tandem mass spectrometry, whereas a whole-blood sample collected in an EDTA tube is drawn for molecular genetic analysis. Both confirmatory biochemical and molecular genetic investigations are performed at the national reference center.

Although 1257 newborns were initially assigned to the risk group, 10 dried blood spot samples were excluded from the final analysis because the material was unsuitable for further molecular testing (e.g., insufficient blood volume or poor sample quality). Consequently, the final cohort examined at the reference center consisted of 1247 newborns. Thus, not all individuals in the risk group were examined, and some cases of hyperphenylalaninemia may have remained undetected. However, the proportion of unexamined cases accounted for less than 1% of the total risk group and therefore did not significantly affect the total number of identified patients, the estimated incidence of HPA in the Russian Federation, or the mutation spectrum in the genes of interest.

At the second stage of neonatal screening, repeat analysis by MS/MS was carried out simultaneously with the detection of 25 common pathogenic variants in the *PAH* gene using allele-specific multiplex ligation-dependent probe amplification (MLPA) [58].

In cases where only one pathogenic variant or no pathogenic/likely pathogenic variants in the *PAH* gene were identified in newborns with elevated phenylalanine levels after retesting, next-generation sequencing (NGS) was carried out using a custom panel including the *PAH*, *PTS*, *QDPR*, *GCH1*, *PCBD1*, *SPR* and *DNAJC12* genes on an Illumina MiSeq platform with the MiSeq Reagent Kit v2 (500 cycles). Library preparation included ultramultiplex PCR followed by sequencing (AmpliSeq™, Illumina, San Diego, CA, USA).

**Figure 6:**
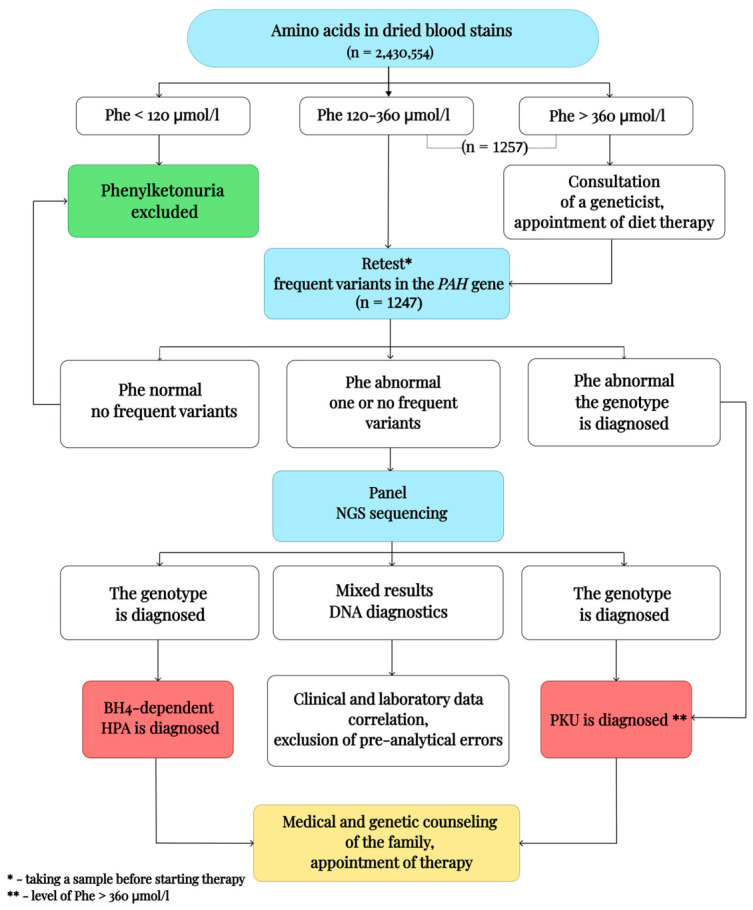
Algorithm for neonatal screening of hyperphenylalaninemia.

At the next stage, for newborns without two pathogenic *PAH* variants but with elevated retest phenylalanine levels, quantitative MLPA analysis was performed using the commercial SALSA MLPA Probemix P055-PAH kit (MRC-Holland, Amsterdam, The Netherlands) to detect gross deletions and duplications in the PAH gene (Figure 6).

Study limitations:

1. In 2023, the first year of the implementation of expanded neonatal screening, the introduction of tandem mass spectrometry (MS/MS) across the regions of the Russian Federation was not simultaneous. In a number of regions, such as the Moscow Oblast, Sverdlovsk Oblast, and Saint Petersburg, biochemical testing (predominantly fluorimetry) was still used in 2023; accordingly, samples from at-risk newborns were not sent to the reference center. The non-simultaneous implementation of MS/MS across regions in 2023, together with temporarily incomplete screening coverage in several regions during the initial phase of the program, should be considered a limitation of this study. However, these factors were unlikely to have substantially affected the calculated nationwide incidence of molecularly confirmed HPA. Our incidence estimate was based on the combined 2023–2024 cohort rather than on the transitional period alone.

The coverage rate of expanded neonatal screening in 2023 was below 85% in three regions, below 90% in three regions, and below 95% in eight regions [59]. According to this official national screening data for 2023, the overall screening coverage in the Russian Federation was 98.03%, corresponding to fewer than 25,000 unscreened newborns nationwide. Given the estimated HPA incidence of 1:4518, this would statistically correspond to only approximately 5–6 potentially missed cases across the country, with an even smaller contribution from the 14 regions where coverage was below 95%. Therefore, these limitations were more likely to influence the detection of borderline or mild cases and some regional comparisons than to substantially bias the overall national incidence estimate.

2. Under the current expanded neonatal screening protocol, family-based segregation analysis (such as trio testing) is not routinely performed. As a result, it was not possible to definitively establish the cis/trans phase of the alleles in newborns carrying two heterozygous variants. Consequently, in cases where two pathogenic variants were identified, a compound heterozygous state was presumed. The lack of formal segregation analysis, however, must be acknowledged as a methodological limitation of this study.
3. Limitation of the existing targeted next-generation sequencing (NGS) panels and standard Sanger sequencing protocols utilized in this study is that they cover only coding exons and immediately adjacent exon-intron boundaries (typically ±20 base pairs). This traditional targeted approach does not allow for the detection of deep intronic variants or complex structural rearrangements in the *PAH* gene, which may be the underlying cause of hyperphenylalaninemia in patients where a second pathogenic variant remains unidentified. To comprehensively address this gap, the entire cohort of 196 patients with a single heterozygous variant is currently undergoing WGS.

Statistical analysis methods:

The incidence of hyperphenylalaninemia per 100,000 individuals (Supplementary Table S2) was calculated as the ratio of newborns with two variants in HPA-associated genes identified through neonatal screening to the total number of screened individuals in a given region:

p = (p_1_/n) × 100,000,

where p_1_ is the number of children diagnosed with PKU/HPA, and n is the total number of observations (i.e., the cohort size).

The standard error (*SE*) was calculated using the following formula:

$$SE=\sqrt{\frac{p(1-p)}{n}\times 100,000}$$

## 5. Conclusions

The implementation of a comprehensive assessment of newborns using tandem mass spectrometry and molecular genetic testing within the framework of expanded neonatal screening in the Russian Federation has demonstrated high diagnostic efficiency. The observed incidence of hyperphenylalaninemia was 1:4518 newborns, compared with the previously estimated frequency of 1:7000 based on screening data prior to 2023. The program enables the detection of mild forms of phenylketonuria, facilitating timely initiation of treatment, and reveals regional differences that allow healthcare professionals to optimize treatment strategies and provide informed counseling to couples planning pregnancy, particularly those with a family history of children affected by hyperphenylalaninemia.

## Figures and Tables

**Table 1:** Genotypes and Phe levels in a risk cohort of 1247 newborns examined in the reference center.

Phe Levels (MS/MS Retest)	Genotype	Number of Newborns (1247 in Total)	Proportion of Risk Group Newborns Sent to the Reference Center
Phe ≥ 120 µmol/L	2 *PAH* variants	496	39.8%
	2 variants in BH4-deficient HPA-causing genes	4	0.3%
	1 variant in the *PAH* gene	37	3.0%
	1 variant in the *PTS* gene	3	0.2%
	1 variant in the *PCBD1* gene	1	0.1%
	No variants in the examined genes	49	3.9%
	1 *PAH* variant and 1 variant in the BH4-deficient HPA-causing genes	1	0.1%
	2 VUS in the *PCBD1* gene	1	0.1%
	Total	592	47.5%
Phe < 120 µmol/L	2 *PAH* variants	38	3.0%
	1 *PAH* variant	150	12.0%
	No variants in the examined genes	459	36.8%
	1 *PAH* variant and 1 variant in the BH4-deficient HPA-causing genes	8	0.6%
	Total	655	52.5%

Note: Regarding the 196 newborns in whom only a single heterozygous *PAH* variant was identified, it is highly likely that we are observing transient hyperphenylalaninemia in healthy carriers. However, the possibility of a second pathogenic variant evading detection by current methods cannot be definitively excluded. Therefore, further investigation of these cases using whole-genome sequencing (WGS) is currently underway.

**Table 3:** Variants detected in BH4-deficient HPA-causing genes.

N	Gene	Variant 1	Variant 2
1	*PTS* (NM_000317.3)	c.216T>A (p.Asn72Lys)	c.317C>T (p.Thr106Met)
2	*PTS* (NM_000317.3)	c.108C>G (p.Asn36Lys)	c.244-1G>T (p.?)
3	*QDPR* (NM_000320)	c.68G>A (p.Gly23Asp)	c.68G>A (p.Gly23Asp)
4	*QDPR* (NM_000320)	c.538_545delinsTACGGGA (p.Leu181ThrfsTer16?)	c.538_545delinsTACGGGA (p.Leu181ThrfsTer16?)

**Table 4:** Median phenylalanine (Phe) levels at primary and repeat blood sampling in groups of newborns with different genotypes.

Genotype	Number of Newborns	Median Phe (µmol/L), First Sampling	Median Phe (µmol/L), Second Sampling	Significance of Differences	Stage 1 of Screening			Stage 2 of Screening		
					<120 (%)	120–360 (%)	>360 (%)	<120 (%)	120–360 (%)	>360 (%)
2 *PAH* variants	503	258.0	288.0	*p* < 0.05	1.2	72.2	26.6	6.8	51.9	41.4
2 pathogenic variants in genes causative of BH4-deficient HPA	4	276.0	648.0	*p* = 0.3125	0.0	75.0	25.0	0.0	25.0	75.0
1 *PAH* variant	182	132.8	79.822	*p* < 0.05	6.0	91.2	2.7	80.2	17.6	2.2
1 pathogenic variant in genes causative of BH4-deficient HPA	4	183.5	198.4	*p* = 0.875	0.0	75.0	25.0	0.0	75.0	25.0
Multiple heterozygous variants in different HPA genes	7	134.0	87.7	*p* < 0.05	0.0	100.0	0.0	100.0	0.0	0.0
No variants in HPA genes	500	132.0	60.1	*p* < 0.05	93.6	0.8	90.4	8.8	0.8	93.6

Note: The analysis of phenylalanine (Phe) concentration dynamics between primary and repeat testing was performed for newborns with paired quantitative data. In 34 children (6.3%), elevated Phe levels (>120 µmol/L) were recorded during primary screening, sufficient to refer for repeat testing. However, the exact numerical values were not documented in the primary medical records, which did not allow for including these cases in the dynamic analysis. Statistical evaluation was performed using the Wilcoxon signed-rank test for paired observations, with a significance threshold of *p* < 0.05.

**Table 5:** Frequency of response to sapropterin (BH4) therapy in Russia and in international studies: number of responders/total number, proportion, and results of comparative analysis (χ^2^, *p*).

Country	Total Number of Responders/Total Number of Patients	Proportion	χ^2^	*p*-Value
Russian Federation	295/534	55%	—	—
Netherlands	108/177	61.0%	1.80	0.179
Italy	20/46	43.5%	2.36	0.124
Japan	59/203	29.1%	40.39	<0.001
China	88/346	25.4%	75.90	<0.001
Spain	102/296	34.5%	32.97	<0.001
Serbia	31/61	50.8%	0.43	0.538

## Data Availability

The original contributions presented in this study are included in the article/Supplementary Materials. Further inquiries can be directed to the corresponding author.

## Supplementary Materials

The following supporting information can be downloaded at: https://www.mdpi.com/article/10.3390/ijms27125597/s1.

## Abbreviations

BH4	Tetrahydrobiopterin
ENS	Expanded neonatal screening
HPA	Hyperphenylalaninemia
MLPA	Multiplex Ligation-dependent Probe Amplification
MS/MS	Tandem mass spectrometry
NGS	Next-generation sequencing
PAH	Phenylalanine hydroxylase
Phe	Phenylalanine
PKU	Phenylketonuria
SE	Standard error
